# Methamphetamine-induced Occludin Endocytosis Is Mediated by the Arp2/3 Complex-regulated Actin Rearrangement*[Fn FN3]^♦^

**DOI:** 10.1074/jbc.M113.483487

**Published:** 2013-09-30

**Authors:** Minseon Park, Hyun-Jung Kim, Brian Lim, Adam Wylegala, Michal Toborek

**Affiliations:** From the ‡ Department of Biochemistry and Molecular Biology, Miller School of Medicine, University of Miami, Miami, Florida 33136 and; the § Department of Neurosurgery, University of Kentucky, Lexington, Kentucky 40536

**Keywords:** Actin, Brain, Cytoskeleton, Endocytosis, Endothelium, Tight Junctions, Methamphetamine, Arp2/3 Complex, Actin, Nucleation, Blood-Brain Barrier, Occludin, Endocytosis, Endo

## Abstract

Methamphetamine (METH) is a drug of abuse with neurotoxic and neuroinflammatory effects, which include disruption of the blood-brain barrier (BBB) and alterations of tight junction protein expression. This study focused on the actin cytoskeletal rearrangement as a modulator of METH-induced redistribution of tight junction protein occludin in brain endothelial cells. Exposure to METH resulted in a shift of occludin localization from plasma membranes to endosomes. These changes were accompanied by activation of the actin-related protein 2/3 (Arp2/3) complex, which stimulates actin polymerization by promoting actin nucleation. In addition, METH-induced coronin-1b phosphorylation diminishes the inhibitory effect of nonphosphorylated coronin-1b on actin nucleation. Blocking actin nucleation with CK-666, a specific inhibitor of the Arp2/3 complex, protected against METH-induced occludin internalization and increased transendothelial monocyte migration. Importantly, treatment with CK-666 attenuated a decrease in occludin levels in brain microvessels and BBB permeability of METH-injected mice. These findings indicate that actin cytoskeletal dynamics is detrimental to METH-induced BBB dysfunction by increasing internalization of occludin.

## Teaser

**Background:** Methamphetamine is a drug of abuse that disrupts the blood-brain barrier.

**Results:** Blocking actin nucleation protects against methamphetamine-induced occludin internalization and disruption of blood-brain barrier integrity.

**Conclusion:** Methamphetamine-induced transendothelial breaches may result from actin-mediated redistribution of occludin.

**Significance:** Actin cytoskeletal dynamics modulates redistribution of occludin and blood-brain barrier integrity.

## Introduction

Methamphetamine (METH)^3^ abuse is one of the fastest growing drug problems, affecting over 35 million users worldwide (1). METH neurotoxicity has been characterized by dysregulated synaptic reuptake of major monoamine neurotransmitters and the generation of oxidative stress (2). METH abuse can also result in vascular function impairments, such as myocardial infarction, stroke, and cardiomyopathy (3–5). In addition, the disruption of the blood-brain barrier (BBB) has been established as one of the most prominent events of METH toxicity (6–8).

The BBB separates brain tissues from the substances circulating in the blood and maintains central nervous system (CNS) homeostasis. The critical elements responsible for BBB integrity are tight junctions (TJs) existing between the neighboring endothelial cells of brain microvessels (9, 10). TJs are constituted by multiple protein components that involve transmembrane proteins (*e.g.* occludin, claudins, and junctional associated molecules) linked to the actin cytoskeleton via interaction with cytoplasmic zonula occludens proteins. Occludin is a 60–65-kDa phosphoprotein that is highly expressed in cerebral endothelium but sparsely distributed in peripheral endothelia (11). It consists of four transmembrane domains, which bind to the two extracellular loops of claudin forming the paracellular component of the TJs. Occludin is anchored to the actin cytoskeleton via its binding to ZO-1 protein. Occludin is highly sensitive to a number of pathological stresses, including oxidative stress, which decreases occludin expression resulting in BBB disruption (12, 13). However, the precise mechanisms regulating occludin protein expression are not fully understood.

The actin cytoskeleton is important for cell shaping, biological cell movements, and cytokinesis. In endothelial cells, the paracellular permeability of the BBB is maintained due to the equilibrium between contractile forces generated by the endothelial cytoskeleton and adhesive forces produced at endothelial cell-cell junctions and cell-matrix contacts (14, 15). Rapid actin assembly and turnover are required for diverse cellular processes. The actin-related protein 2/3 (Arp2/3) complex is one of the principal actin-polymerizing and -organizing factors (16, 17). It works by initiating actin filament branches on the sides of existing mother filaments and cross-linking filaments into Y-branched networks in a process called nucleation (18). Activity of the Arp2/3 complex is stimulated by several regulatory proteins, including Wiskott-Aldrich syndrome protein (WASp) (19). In contrast, coronin-1b is an inhibitory factor, which blocks actin nucleation by binding to the activated Arp2/3 complex (20). This inhibitory effect can be prevented by coronin-1b phosphorylation, providing an additional element of complex regulatory machinery, thereby tuning the cellular cytoskeleton in response to cellular stressors.

The role of actin cytoskeleton rearrangement in drug-induced BBB dysfunction is poorly understood. This study provides evidence that METH stimulates actin rearrangement by activation of the Arp2/3 complex, resulting in occludin endocytosis and transendothelial monocyte migration.

## EXPERIMENTAL PROCEDURES

### 

#### 

##### Cell Culture and Treatment Factors

Immortalized human brain microvascular endothelial cells (hCMEC/D3 cells) (21) were cultured in EBM-2 medium, supplemented with EGM-2 Bullet kit, which contains insulin-like growth factor-I (IGF-I), epidermal growth factor (EGF), basic fibroblast growth factor, vascular endothelial growth factor (VEGF), hydrocortisone, ascorbate, gentamycin, and fetal bovine serum (FBS) (Lonza, Walkersville, MD). All cell culture plates were coated with 150 μg/ml of rat tail collagen type I (BD Biosciences) for 1 h. Methamphetamine hydrochloride (US Pharmacopeia, Rockville, MD) was dissolved in distilled water and added directly into brain endothelial cells in a final concentration of 10 μm. This level is a typical METH plasma concentration in abusers (22). In addition, we demonstrated in a series of dose-dependent experiments that METH at this concentration can disrupt TJ proteins and decrease occludin levels (23). In selected experiments, cultures were treated with U0126 (1 μm; Sigma) to inhibit the MAPK signaling pathway or with CK-666 (80 μm; Tocris Bioscience, Ellisville, MO) to block the Arp2/3 complex. The inhibitors were added 1 h before METH exposure and maintained in cell culture media for the duration of METH treatment.

##### Endocytosis Assay

Confluent cultures were biotinylated with 0.5 mg/ml sulfo-NHS-SS-Biotin (Pierce) in phosphate-buffered saline containing 0.9 mm CaCl_2_ and 0.33 mm MgCl_2_ (PBS/CM) at 4 °C for 30 min. After free biotin was quenched with 50 mm NH_4_Cl in PBS/CM at 4 °C for 15 min, the samples were incubated with pre-warmed media containing METH or vehicle at 37 °C for the indicated times to allow endocytosis. Biotins remaining on the cell surface were then stripped off with 50 mm MESNA in 100 mm Tris/HCl (pH 8.6) containing 100 mm NaCl and 2.5 mm CaCl_2_ at 4 °C for 30 min and quenched with 5 mg/ml iodoacetamide in PBS/CM at 4 °C for 15 min. After lysis with RIPA buffer (10 mm sodium phosphate (pH 7.2), 150 mm NaCl, 1% Triton X-100, 0.5% sodium deoxycholate, and 0.1% SDS), biotinylated occludin was isolated with UltraLink Immobilized NeutrAvidin Plus beads (Pierce), and the levels were analyzed by immunoblotting.

##### Sucrose Gradient Analysis

Sucrose gradient analysis was performed as described previously with minor modification (24). Cells were lysed in 1 ml of ice-cold 1% (v/v) Triton X-100 in MNE buffer (20 mm MES, pH 6.5, 150 mm NaCl, 5 mm EDTA) in the presence of proteinase and phosphatase inhibitor mixture (Roche Applied Science) for 30 min on ice. Lysates were homogenized on ice with 20 strokes of a Dounce homogenizer and centrifuged for 10 min at 2,000 rpm at 4 °C. Supernatants were then diluted 1:1 with 80% (w/v) sucrose in MNE buffer, placed at the bottom of a 14 × 89-mm ultracentrifuge tube (Beckman Coulter, Brea, CA), and overlaid gently with 4 ml of 35% and 4 ml of 5% sucrose. The resulting 5–40% discontinuous sucrose gradient was centrifuged at 35,000 rpm for 20 h in a swinging bucket rotor (model SW40, Beckman Instruments) at 4 °C to separate the low density lipid rafts. Fractions (1 ml) were collected from the top to the bottom and analyzed by immunoblotting.

##### Immunofluorescence

To stain for occludin and Rab7, monolayers of brain endothelial cells grown on type I collagen-coated glass coverslips were fixed in 4% paraformaldehyde for 15 min and permeabilized for 10 min in PBS. Nonspecific binding was blocked with 3% bovine serum albumin (BSA; Boston BioProducts) in PBS for 1 h. Coverslips were then incubated overnight at 4 °C in a humidified atmosphere with anti-occludin antibody conjugated with Alexa Fluor® 594 and goat anti-Rab7 antibody diluted at 1:100 in PBS containing 3% BSA. After three washes with PBS, the coverslips were incubated with secondary Alexa Fluor® 488 donkey anti-goat antibody (Invitrogen) at 1:200 in PBS containing 3% BSA for 1 h, washed repeatedly with PBS, and mounted with ProLong Gold Antifade reagent containing 4′,6-diamidino-2-phenylindole (DAPI) to visualize the nuclei (Invitrogen). The images were captured by a confocal microscope (Olympus Fluoview V5; Olympus America, Melville, NY) using constant contrast and brightness conditions. To quantify the overall F-actin, cultures grown on the glass coverslips were fixed and permeabilized as described above, followed by incubating with Alexa Fluor® 594 or 488 phalloidin (Invitrogen) at 1:100 diluted in PBS for 1 h. Fluorescence was captured under the constant contrast and brightness conditions by a fluorescence microscope (Eclipse Ti; Nikon Instruments, Melville, NY), and the intensity was measured using the program provided by the manufacturer. At least six images from three independent experiments were quantified. Semi-quantitative analyses of F-actin intensity between the neighboring nuclei were measured using a confocal microscope.

##### Immunoblotting

Cells were washed with PBS and lysed for 30 min on ice in Nonidet P-40 lysis buffer containing 25 mm HEPES (pH 7.4), 150 mm NaCl, 4 mm EDTA, 1% Nonidet P-40 (Roche Applied Science) containing proteinase and phosphatase inhibitor mixture (Roche Applied Science). Lysates were centrifuged at 14,000 × *g* for 10 min, and the supernatants were used for immunoblotting. Protein concentrations were determined using BCA protein assay kit (Thermo Scientific, Rockford, IL). Samples were separated on SDS-polyacrylamide gels, transferred onto PVDF membranes (Bio-Rad), and incubated with the respective antibodies. The antibody against occludin was obtained from Zymed Laboratories Inc., and the rabbit polyclonal antibodies against phosphorylated ERK1/2 or total ERK1/2 were purchased from Cell Signaling Technology (Danvers, MA). The Arp2/3 complex regulation antibody sampler kit containing polyclonal anti-Arp2, WASp/N-WASp, coronin-1b, and phospho-specific coronin-1b antibodies was obtained from ECM Biosciences (Versailles, KY). The mouse monoclonal antibody against early endosome antigen 1 (EEA1) was from BD Biosciences. The goat polyclonal anti-Rab7 and polyclonal anti-GAPDH antibodies conjugated with peroxidase were purchased from Santa Cruz Biotechnology. Immunoblots were visualized using the ECL detection system (Amersham Biosciences).

##### Immunoprecipitation

The cells were lysed in Nonidet P-40-lysis buffer containing freshly added protease and phosphatase inhibitor mixture solution as described above. Total protein lysates (500 μg) were incubated with 1 μg of anti-Arp2 antibody for 16 h at 4 °C with gentle rotation. Then 30 μl of 50% slurry of protein A/G-agarose (Santa Cruz Biotechnology) was added into each lysate, incubated for an additional hour, and centrifuged at 5,000 rpm for 3 min to collect immune complexes. The remaining supernatants were saved and analyzed for GAPDH levels as an internal control. The immunoprecipitates were washed three times with the lysis buffer and analyzed by immunoblotting.

##### Coronin-1b Silencing

Coronin-1b silencing was performed as described previously (25) using small interfering RNA (siRNA) targeting human coronin-1b (Santa Cruz Biotechnology). Silencer Negative Control 1 siRNA (Applied Biosystems, Austin, TX) was used as nonspecific control siRNA. Brain endothelial cells were transfected overnight with 100 nm of coronin-1b or control siRNA using GeneSilencer siRNA transfection reagent (Genlantis, San Diego). Cells were then washed and allowed to recover for 2 days in normal medium before METH exposure. The effectiveness of coronin-1b silencing was confirmed by probing for coronin-1b levels in the supernatants obtained after precipitation of immune complexes.

##### Monocyte Transmigration Assay

Monocyte transendothelial migration assay was performed as described previously (23). Cells were seeded at the density of 2 × 10^5^ cells per insert on rat tail collagen type I-coated Transwell Permeable Supports (1.12 cm^2^ diameter, 3.0 μm pores; Corning) and cultured for 3 days. The cells were serum-depleted for 24 h before the assay. U937 human leukemic monocyte lymphoma cells were labeled with calcein-AM (Invitrogen) and added onto endothelial monolayers in the amount of 1 × 10^5^ cells per insert. The co-cultures were treated with METH (10 μm) or vehicle for 5 h. Then the fluorescence intensity was assessed in aliquots collected from the lower chamber of the Transwell system as the indicator of labeled monocytes transmigrating across endothelial monolayers from the upper to the lower chamber. Fluorescence analyses were performed using SpectraMax Gemini EM Fluorescence Microplate Reader with SoftMax Pro software (Molecular Devices, Sunnyvale, CA).

##### Animals, METH Exposure, and Isolation of Brain Microvessels

All animal procedures were approved by the University of Miami Institutional Animal Care and Use Committee in accordance with National Institutes of Health guidelines. Male C57BL/6 mice (Charles River Laboratories, Wilmington, MA), 12–13 weeks of age, were weight-matched and randomly assigned to various treatment groups. Mice were injected intraperitoneally with 5 mg/kg CK-666 dissolved in 40% (v/v) dimethyl sulfoxide (DMSO) for 30 min before a single dose of METH injected intraperitoneally (10 mg/kg). 40% (v/v) DMSO or saline (100 μl/mouse) was used as a vehicle control for CK-666 or METH, respectively. Animals were anesthetized and perfused with saline 1 h post-METH administration. As the BBB is formed at the level of cerebral microvessels, the microvessels were isolated from brains as described previously (26). Briefly, brains were removed after transcardial perfusion and immediately immersed in ice-cold PBS. Choroid plexus, meninges, cerebellum, and brain stem were removed, and brains were homogenized in isolation buffer (102 mm NaCl, 4.7 mm KCl, 2.5 mm CaCl_2_, 1.2 mm KH_2_PO_4_, 1.2 mm MgSO_4_, 15 mm HEPES, 25 mm NaHCO_3_, 10 mm glucose, 1 mm sodium pyruvate with proteinase inhibitors). Then 26% dextran (*M*_r_ 150,000) in isolation buffer was added, and samples were centrifuged (5,800 × *g*; 4 °C) for 20 min. The supernatants were discarded; pellets were resuspended and filtered through a 120-μm mesh filter. Filtered homogenates were re-pelleted by centrifugation (1,500 × *g*; 4 °C) for 10 min and either smeared onto a glass microscope slide for fluorescence microscopy analysis or resuspended in lysis buffer for analysis of protein expression.

##### Occludin Assessment in Brain Microvessels

Freshly isolated microvessels were spread onto glass microscope slides and heat-fixed for 10 min at 95 °C, followed by treatment with 4% paraformaldehyde for 10 min at room temperature. They were then washed three times with PBS and permeabilized in PBS containing 0.1% Triton X-100 for 10 min. Microvessels sized between 4 and 8 μm in diameter were selected for analyses. Occludin immunoreactivity was determined using anti-occludin antibody conjugated with Alexa Flour® 594 (Invitrogen) diluted at 1:100 with 3% BSA in PBS. In addition, the microvessels were co-stained for CD31/PECAM-1 expression, a marker for the vascular endothelium (supplemental Fig. 1). Slides were mounted with ProLong Gold Antifade reagent containing DAPI (Invitrogen). Images were acquired using a fluorescence microscope and analyzed with software provided by the manufacturer (Nikon). At least 26 different microvessel images per each group were used for the quantification.

##### BBB Permeability Assay

BBB permeability was measured as described previously with modifications (27). Mice exposed to METH and/or vehicle were injected intraperitoneally with 200 μl of 10% sodium fluorescein (NaFl; Sigma). Twenty minutes later, the animals were anesthetized with isoflurane in oxygen; blood was collected via heart puncture, and the mice were transcardially perfused with saline to remove blood from the intravascular compartment. Our previous study indicated that the hippocampus is most sensitive to METH toxicity and BBB disruption (27); therefore, these regions were dissected, homogenized in 100 μl of RIPA buffer, and centrifuged at 14,000 × *g* for 10 min. Fifty microliters of the clarified supernatants were transferred into 60 μl of 10% trichloroacetic acid (TCA) and centrifuged at 1,000 × *g* for 10 min. Then 100 μl of supernatant aliquots were mixed with 8.33 μl of 5 m NaOH to neutralize TCA, and fluorescence was measured with SpectraMax Gemini EM microplate fluorescence reader (Molecular Device). BBB permeability was expressed as NaFl (in picograms) in the hippocampus/protein (in micrograms) and normalized to its levels in serum.

##### Statistical Analysis

The data were analyzed using GraphPad Prism software, and experimental treatments were compared pairwise with control treatments using two-way analysis of variance followed by Tukey's multiple comparisons test or Fisher's least significant difference. A value of *p* < 0.05 was considered significant.

## RESULTS

### 

#### 

##### METH Enhances Occludin Endocytosis

**FIGURE 1.**
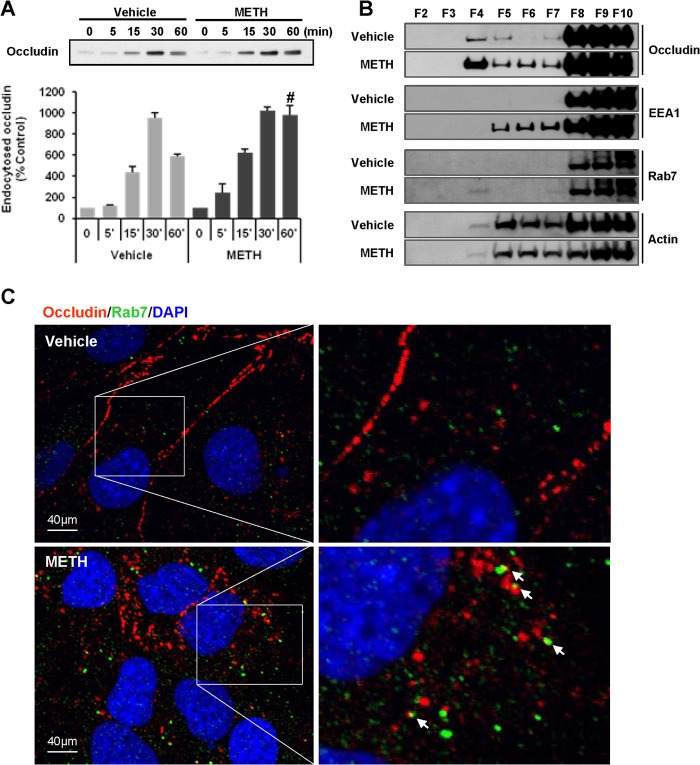
**METH enhances occludin endocytosis.** *A,* endocytosis assay was performed by biotinylation of brain endothelial cells with sulfo-NHS-SS-biotin. After METH exposure for the indicated time, the biotinylated cell surface was stripped off and endocytosed. Biotinylated occludin was isolated followed by immunoblotting. The image reflects representative data of three independent experiments, and the *bar graph* shows quantitative results (mean ± S.E.) from these experiments. #, *p* < 0.05 compared with vehicle at the corresponding time. *B,* cellular distribution of occludin, EEA1, and Rab7 was examined by immunoblotting after sucrose density ultracentrifugation in control and METH-exposed (10 μm for 1 h) brain endothelial cells. Actin was determined as a loading control. *C,* immunofluorescence of occludin (*red*) and Rab7 (*green*) in METH-exposed (10 μm for 1 h) brain endothelial cells. Regions of co-localization of occludin and Rab7 are depicted in *yellow* and indicated by *arrows*.

Although TJs are critical structures maintaining the integrity of the brain endothelium, there are cyclic movements of TJ proteins between the cell membrane and other cellular compartments. We evaluated intracellular occludin movements by biotinylating cell surface proteins. In control cultures of brain endothelial cells, the levels of endocytosed occludin increased linearly for up to 30 min, followed by a decrease between 30 and 60 min. Treatment with 10 μm METH disrupted this cycle by enhancing endocytosis. In contrast to control treatment, the levels of endocytosed occludin in METH-exposed cultures did not diminish between 30 and 60 min but rather remained significantly elevated (Fig. 1*A*).

To further evaluate to which of the cellular compartments endocytosed occludin transfers as the result of METH exposure, sucrose density ultracentrifugation of cellular fractions was performed, followed by determination of occludin, EEA1 (early endosome antigen1; an early endosome-associated protein) (28), and Rab7 (a small GTPase localized to the late endosomes) (Fig. 1*B*) (29). Actin was determined as a loading control. Within 1 h of exposure to 10 μm METH, the EEA1 immunoreactivity was increased in fractions 5–7, and the Rab7 immunoreactivity appeared in fraction 4. Interestingly, METH exposure increased occludin immunoreactivity in fractions 4–7, further suggesting that METH exposure enhances occludin endocytosis.

We then confirmed this phenomenon by immunostaining cells with occludin and Rab7 (Fig. 1*C*). In control cells, most occludin immunoreactivity was observed in the regions corresponding to cell membranes, and Rab7 immunostaining was scattered in the cytoplasm. In cells exposed to METH (10 μm) for 1 h, occludin immunoreactivity was fragmented and scattered. In contrast, Rab7 immunoreactivity was enhanced and exhibited a prominent co-localization with occludin (Fig. 1*C, arrows*). These observations indicate that occludin is internalized from the cell surface after METH exposure and then translocalized to late endosomes within an hour.

##### METH Stimulates Actin Polymerization

Actin polymerization is involved in various cellular processes. Cells have a substantial pool of monomeric actin able to quickly polymerize to form helical actin filaments (F-actin) and reorganize the cytoskeleton when subjected to environmental stress. F-actin can be bundled by binding protein into thick actin structures called stress fibers (30). There also appears to be a close functional link between the actin cytoskeleton and the internalization step of endocytosis (31–33).

**FIGURE 2.**
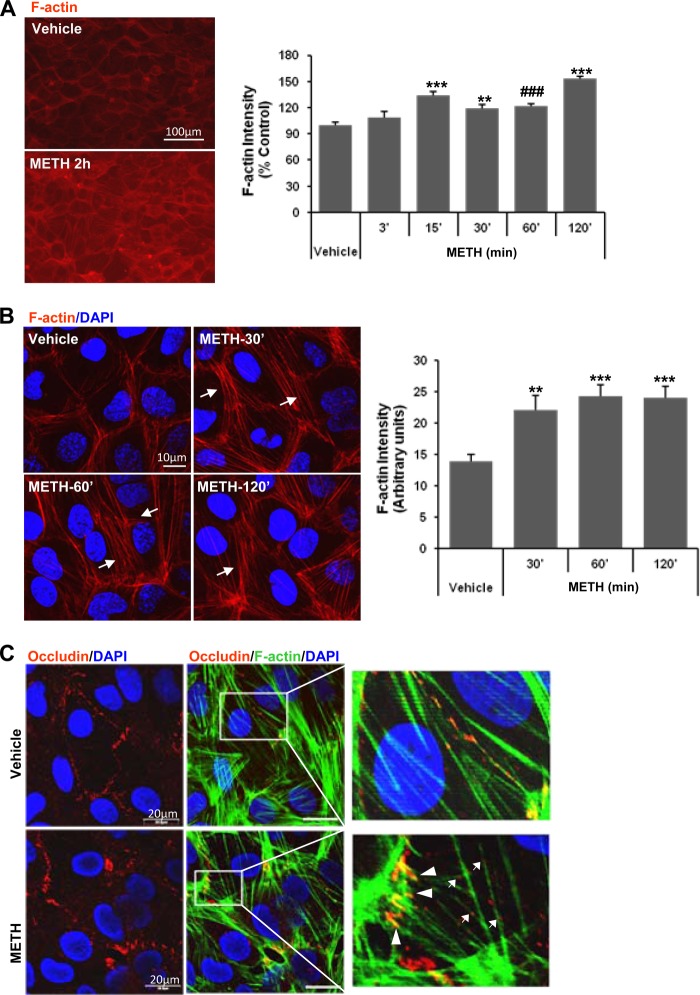
**METH increases actin polymerization and alters occludin-actin localization.** *A,* brain endothelial cells were exposed to 10 μm of METH for the indicated time, followed by F-actin staining with Alexa Fluor® 594 phalloidin. *Left panel* indicates representative images, and the fluorescence intensity was quantified in the form of a *bar graph* (*right panel*). *Bar graph* depicts the mean ± S.E. Data are statistically significant as compared with vehicle at **, *p* = 0.003; ###, *p* = 0.0003, and ***, *p* = 0.0001. *B,* cells were treated with METH (10 μm) for the indicated time period, followed by staining of F-actin as in *A*, and analysis under confocal microscope. Unit intensity of F-actin was obtained by dividing the total actin fluorescence intensity between the neighboring nuclei by the distance. Actin fluorescence was measured in over 30 cells in each group using six independent cultures and quantified in the form of a *bar graph. Bar graph* depicts the mean ± S.E. Statistically significant as compared with vehicle at **, *p* = 0.002, and ***, *p* = 0.0001. *C,* interaction of occludin with F-actin. Cells were exposed to 10 μm METH for 1 h. F-actin was stained with Alexa Fluor® 488 phalloidin (*green*); occludin was immunostained with anti-occludin antibody (*red*), and nuclei were visualized by DAPI (*blue*). The *insets* illustrate areas of distinct binding between occludin and F-actin in control and METH-exposed cells.

To investigate the involvement of actin polymerization in METH-induced cellular effects, brain endothelial cells were exposed to METH (10 μm) for up to 2 h, followed by labeling F-actin with Alexa Fluor® 594-phalloidin. Fluorescence intensity of F-actin gradually increased with exposure time to METH and reached the maximum level at 2 h post-treatment (Fig. 2*A*). Further analysis of the structural changes of F-actin under a confocal microscope revealed that METH exposure caused a rearrangement of the actin network into thick bundles of stress fibers (*arrows*) (Fig. 2*B, image*). Semi-quantitative analyses of pixel intensity corresponding to F-actin performed across the cells confirmed the increase in F-actin fluorescence intensity by METH exposure (Fig. 2*B, bar graph*). Co-staining for occludin and F-actin identified that occludin is bound to actin filaments both in vehicle- and METH-exposed cells (Fig. 2*C*). Although in control cells these interactions appeared to be uniformly aligned, exposure to METH resulted in increased co-staining for occludin and F-actin in the regions corresponding to tricellular junctions (Fig. 2*C, arrowheads*) and in cytoplasm (*arrows*). These results suggest that METH-induced actin polymerization is closely associated with alterations of intracellular occludin movements.

##### METH Activates Arp2/3 Complex by Increasing Coronin-1b Phosphorylation

**FIGURE 3.**
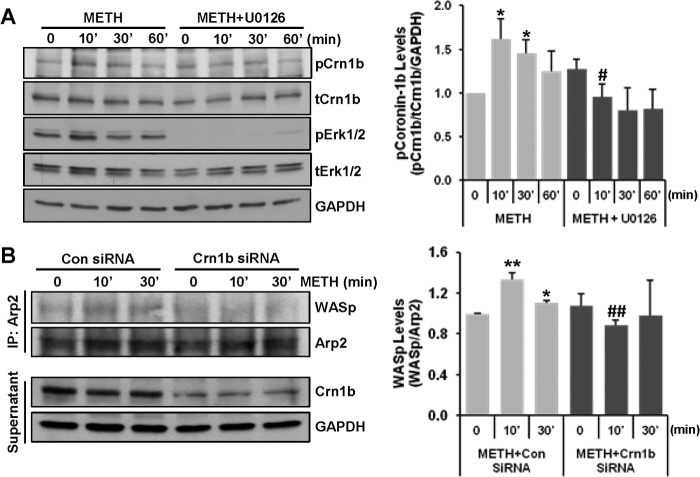
**METH activates the Arp2/3 complex by increasing coronin-1b phosphorylation.** *A,* brain endothelial cells were preincubated with 1 μm U0126, a MEK inhibitor for 1 h, followed by exposure to 10 μm METH for the indicated time. Total coronin-1b (*tCrn1b*), phosphorylated Crn1b (*pCrn1b*), total ERK1/2 (*tERK1/2*), phosphorylated ERK1/2 (*pERK1/2*), and GAPDH (internal control) were detected by immunoblotting. The *blots* are representative images of three experiments, and the *bar graphs* depict the mean ± S.E. of the densitometric analyses of target proteins normalized to GAPDH levels. *, *p* = 0.025 compared with vehicle control, and #, *p* = 0.024 compared with METH at the corresponding time. *B,* brain endothelial cells were transfected with control (*Con*) or coronin-1b (*Crn1b*) siRNA 3 days before 10 μm METH exposure for the indicated time period. Arp2 was immunoprecipitated (*IP*) and probed for WASp. WASp levels were normalized to the precipitated Arp2 protein levels. Images show representative blots from three independent experiments, and the *bar graphs* depict the mean ± S.E. *, *p* = 0.015, and **, *p* = 0.001 compared with vehicle control; ##, *p* = 0.002 compared with *Con siRNA* + *METH* at the corresponding time.

The Arp2/3 complex, which stimulates actin nucleation, has emerged as a central effector of actin polymerization (34). Among various modulating proteins known to regulate Arp2/3 complex activity, coronin-1b has been shown to inhibit the Arp2/3 complex activity (20, 35). Because this inhibitory function can be deterred by phosphorylation of coronin-1b at serine 2 (36), we examined the effects of METH treatment on coronin-1b expression and phosphorylation status. The experiments also involved the assessment of ERK1/2 phosphorylation because this signaling cascade is detrimental to alterations of occludin expression (21, 23). As shown in Fig. 3*A*, coronin-1b was phosphorylated as quickly as 10 min after METH exposure and gradually returned to base line within 1 h. Pretreatment with U0126 (a MEK inhibitor, 1 μm) effectively prevented coronin-1b phosphorylation, indicating dependence on ERK1/2 signaling pathway.

To further address the role of coronin-1b in METH-induced Arp2/3 complex activity, cells were transfected with coronin-1b-specific or control siRNA followed by exposure to METH (10 μm) for up to 30 min. Cells were then lysed, immunoprecipitated with anti-Arp2 antibody, and probed for the activating protein, WASp. METH significantly increased the binding of WASp to Arp2 as early as 10 min post-exposure (Fig. 3*B*). Coronin-1b silencing protected against this effect, suggesting that METH-induced binding between Arp2 and WASp is dependent on the presence of coronin-1b. Thus, METH stimulates the Arp2/3 complex by increasing coronin-1b phosphorylation and binding with WASp.

##### Inhibition of Arp2/3 Complex Protects against METH-induced Alterations of Occludin Levels and Transendothelial Migration of Monocytes

**FIGURE 4.**
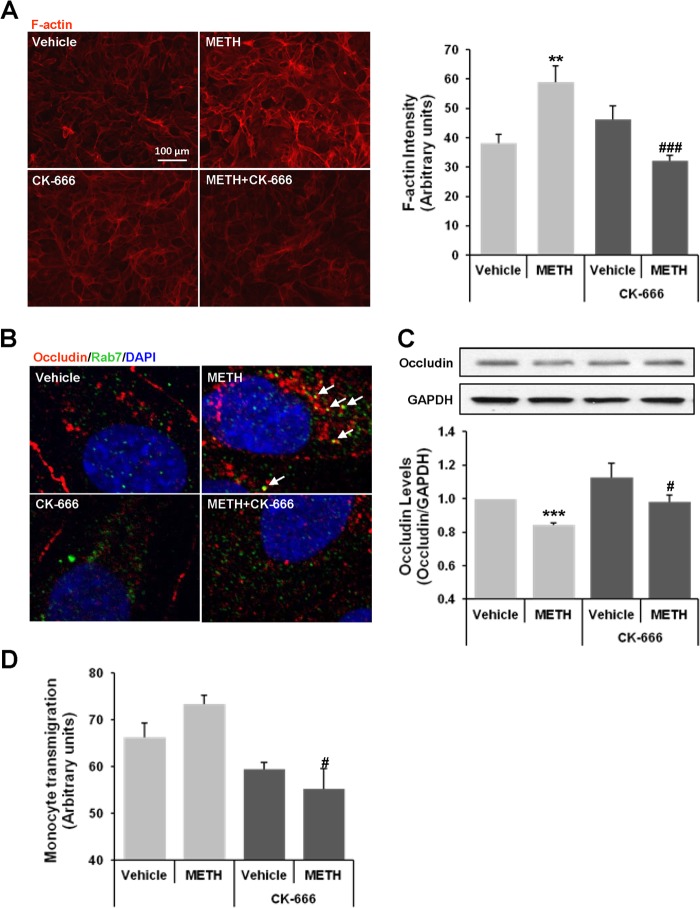
**Inhibition of the Arp2/3 complex protects against METH-induced actin polymerization, alterations of occludin expression, and transendothelial migration of monocytes.** *A,* brain endothelial cells were pretreated with CK-666 (80 μm for 1 h) followed by exposure to 10 μm METH for 1 h. CK-666 was maintained in cell culture media for the duration of METH exposure. F-actin was stained with Alexa Fluor® 594 phalloidin followed by analysis as in Fig. 2. *Left panel* shows representative image from four experiments, and the quantitative data are presented in a *bar graph* (*right panel*). Data are mean ± S.E. **, *p* = 0.004 compared with vehicle control, and ###, *p* = 0.0003 compared with METH. *B*, cells were pretreated with CK-666 as in *A*, followed by exposure to METH for 1 h and followed by immunostaining with occludin (*red*) and Rab7 (*green*). *Arrows* indicate co-localization of occludin and Rab7. *C,* cells were pretreated with CK-666 as in *A*, followed by exposure to METH for 3 h and occludin immunoblotting. The blots reflect representative data from four different experiments, and the *bar graphs* (mean ± S.E.) represent quantified results from these experiments as analyzed by ImageJ and normalized to GAPDH. ***, *p* = 0.0001 compared with vehicle, and #, *p* = 0.018 compared with METH at the corresponding exposure time. *D,* confluent brain endothelial cells grown on transwell inserts were pretreated with CK-666 as in *A* and exposed for 5 h to 10 μm METH in co-cultures with calcein-AM-labeled U937 cells. Data are mean ± S.E. #, *p* = 0.018 compared with METH.

To evaluate whether the Arp2/3 complex mediates METH-induced F-actin polymerization, the cells were pretreated for 1 h with CK-666 (80 μm), an inhibitor of the Arp2/3 complex, followed by a 1-h exposure to METH in the presence of the inhibitor. Fig. 4*A* indicates that inhibition of the Arp2/3 complex activity protects against METH-induced enhanced F-actin formation, demonstrating the dependence of METH-induced actin polymerization on Arp2/3 complex activity. Next, we evaluated a possible link between Arp2/3 complex-mediated actin polymerization and occludin endocytosis in METH-exposed brain endothelial cells. Cells were pretreated with CK-666, followed by exposure to METH (10 μm) for 1 h and immunostaining for occludin and Rab7. Confirming the results from Fig. 1*C*, exposure to METH induced interaction of occludin with Rab7 (*arrows*); however, CK-666 pretreatment protected against this effect (Fig. 4*B*). Because Rab7 was shown to be a necessary component for the transfer of cargo from the late endosome to the lysosome (37), our observations of co-localization between occludin and Rab7 after 1 h of METH exposure suggest a possible degradation of occludin as the result of METH treatment. To address this possibility, cells were exposed to METH (10 μm) for 3 h, followed by immunoblotting for occludin (Fig. 4*C*). METH exposure significantly reduced the levels of occludin; this effect was protected by inhibition of Arp2/3 complex activity. Because the integrity of occludin is important for the barrier functions of brain endothelial cells (23), we also determined the involvement of Arp2/3 complex in METH-induced monocyte migration across a monolayer of brain endothelial cells. Inhibition of the Arp2/3 complex protected against the METH-stimulated transendothelial monocyte passage (Fig. 4*D*), demonstrating that the Arp2/3 complex is involved in METH-induced barrier dysfunction of brain endothelial cells.

##### METH-induced Alterations of Occludin Expression in Brain Microvessels and Disruption of the BBB Integrity Are Attenuated by Inhibition of the Arp2/3 Complex

**FIGURE 5.**
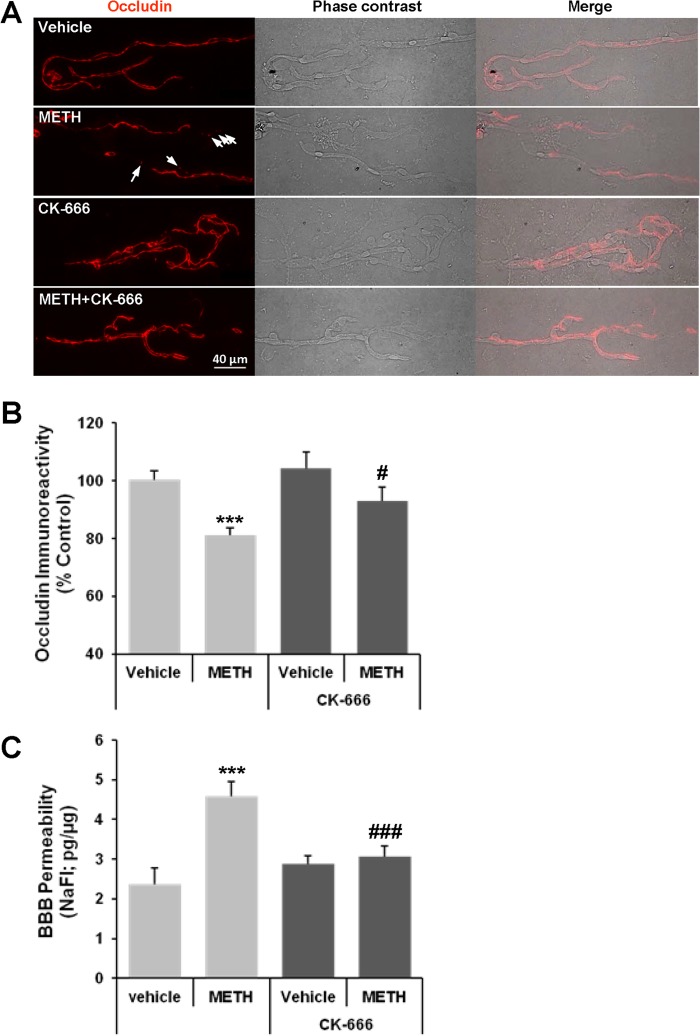
**Inhibition of the Arp2/3 complex protects against METH-induced alterations of occludin levels in brain microvessels and increased BBB permeability.** Mice were pretreated for 30 min with CK-666 (*CK*; 5 mg/kg; intraperitoneally) followed by a 1-h treatment with METH (10 mg/kg; intraperitoneally) and isolation of brain microvessels. *A,* representative images of occludin immunoreactivity (red staining, *left panel*) in isolated brain microvessels. *Arrows* indicate the areas corresponding to the disrupted occludin immunoreactivity. Phase contrast micrographs (*middle panel*) illustrate the isolated microvessels, and merged micrographs (*right panel*) show localized occludin immunoreactivity within the microvessels. *B,* occludin immunofluorescence was assessed in brain microvessels in four mice per group. The obtained results were quantified (mean ± S.E.) and represented in a form of a *bar graph*. ***, *p* = 0.0001 compared with vehicle, and #, *p* = 0.039 compared with METH. *C,* CK-666 protects against METH-induced disruption of the BBB integrity. The data are mean ± S.E., *n* = 6–7. ***, *p* < 0.001 compared with vehicle, and ###, *p* = 0.003 compared with METH.

In the last series of experiments, we employed an *in vivo* mouse model of acute METH abuse. Mice were pretreated with CK-666 (5 mg/kg; intraperitoneally) for 30 min to inhibit the Arp2/3 complex, followed by METH injection (10 mg/kg; intraperitoneally). Control mice were injected with vehicle. Co-staining for CD31/PECAM-1 (supplemental Fig. 1) was performed as a marker of brain endothelial cells. Microvessels isolated from control mice were characterized by relatively continuous staining of occludin. In contrast, a spot-like staining of occludin and a decrease in occludin protein levels were observed in microvessels isolated from METH-exposed mice (Fig. 5*A*, *arrows*). Importantly, pretreatment with CK-666 restored the integrity of occludin immunoreactivity and its levels to control values (Fig. 5*B*), confirming the results from cultured endothelial cells that the Arp2/3 complex plays a critical role in METH-induced occludin alterations.

Next, we evaluated the impact of CK-666 on METH-induced BBB permeability. Because of regional differences in METH-induced impact on brain microvasculature (27, 38), our analyses focused on the hippocampus as the brain region particularly susceptible to METH toxicity (27, 39). As shown in Fig. 5*C*, METH administration resulted in the disruption of the BBB. Importantly, this effect was prevented by CK-666 pretreatment, further confirming the impact of Arp2/3 complex-related actin polymerization on METH-induced cerebrovascular toxicity.

## DISCUSSION

Although disruption of the BBB has been established as one of the critical components contributing to METH-induced neuroinflammation, the role of the actin cytoskeleton, which represents a contractile force in the junctional endothelial cells, in this process has been poorly understood. TJs are constituted by several transmembrane and cytoplasmic proteins; however, it appears that alterations in occludin levels, even without changes in other TJ proteins, are not compensated for and can impact the proper functions of BBB in response to various extracellular stimuli, leading to disruption of barrier functions (12, 40–43). Therefore, the objective of this study was to investigate the relationship between METH-induced actin cytoskeletal rearrangement and occludin alterations.

Important results of this study indicate that occludin is internalized within 1 h of METH exposure, when it partially co-localizes with the markers for early (EEA1) and late endosomes (Rab7). These observations are consistent with literature reports, which describe this event in endothelial cells treated with the pro-inflammatory chemokine CCL2 (24) or VEGF (44), and in epithelial cells exposed to EGF (41). Moreover, occludin and claudin-5 internalization was also linked to loss of function of the brain endothelial barrier as estimated by transendothelial electrical resistance (24). These findings support our observations that increased BBB permeability is associated with redistribution of TJ proteins, with occludin playing a major role in this process.

The fate of internalized TJ proteins varies from degradation to recycling processes. Several publications have demonstrated that occludin can be ubiquitinated, followed by degradation through the ubiquitin-proteasome system in epithelial (45, 46) and endothelial cells (44, 47). Our data indicate that METH exposure results in reduction of the occludin level, which suggests that internalized occludin is degraded; this observation is consistent with co-localization of occludin with Rab7, the marker of late endosomes. Nevertheless, we also observed that the fraction of occludin co-immunostained with Rab11, a recycling endosome marker protein (supplemental Fig. 2), which opens the possibility of occludin availability for recycling back to the cell surface.

The precise mechanisms linking disruption of the BBB integrity to the alterations of actin cytoskeleton are not fully understood. Our data show that METH exposure increases actin polymerization as an early event contributing to its toxicity. Importantly, occludin immunoreactivity is associated with F-actin, and inhibition of actin nucleation blocks occludin endocytosis. Based on these observations, we suggest that METH exposure stimulates reorganization of actin structure, resulting in changes in the cell cytoskeleton's architectural integrity and the translocation of occludin from the membranes to the cytoplasmic compartments via endocytosis. To support a role of actin polymerization in endocytosis, it was demonstrated that actin appears in the plasma membrane during the formation of the primary endocytic vesicles and that dynamic actin structures associate with motile endosomes (48). Indeed, inhibition of actin polymerization using latrunculin-A, a drug sequestering actin monomers, resulted in a strongly impaired endosome mobility in yeast (49) and HeLa cells (50). Inhibition of actin dynamics also blocked redistribution of TJ proteins and protected against increased endothelial cell permeability in acute ischemic conditions (51). Moreover, phosphorylation of myosin light chains (MLC) was demonstrated to regulate TJs in response to diverse physiological and pathophysiological stimuli, including METH (13, 52).

The role of contractile proteins in the regulation of endothelial cell barrier function was previously evaluated in the aspect of MLC phosphorylation. It was shown that once MLC is phosphorylated, it stimulates endothelial cell contraction and results in barrier dysfunction (53). Moreover, constitutively active MLC kinase overexpressed in Caco-2 cells can reorganize F-actin and increase size-selective TJ permeability (54). Our present results indicate that stimulation of actin reorganization can also induce dysfunction of the endothelial barrier integrity via redistribution of cellular occludin.

Novel results in this study indicate that binding of WASp to the Arp2/3 complex is involved in METH-induced actin polymerization. Activators of the Arp2/3 complex, termed the nucleation-promoting factors, are required for the proper spatial and temporal control of actin assembly in cells. Mammalian cells express several nucleation-promoting factors, including WASp and N-WASp. The CA motif of VCA region (verpolin homology region, cofilin homology region, and acidic region) in WASp binds to the Arp2/3 complex, and the V motif binds actin monomers, tethering them to the complex (55–57). In mammalian cells, N-WASp and the Arp2/3 complex are found at sites of clathrin-mediated endocytosis (58), and cells lacking functional N-WASp exhibit reduced internalization kinetics of EGF receptor (59). The WASp family is activated by Rho GTPases like Cdc42 and Rac (60), which correspond with our observations that METH activates Rac1 in brain endothelial cells (23). This suggests that METH-induced GTPase activity may trigger WASp activation followed by promoting Arp2/3 complex-related nucleation and occludin redistribution.

Interestingly, METH-induced actin polymerization was associated with the appearance of stress fibers. Although it is generally accepted that stress fiber assembly is associated with activation of RhoA (61) and mediated in part by phosphorylated MLC, recent evidence indicated that Arp2/3 complex-nucleated actin bundles during transverse arcs are formed by endwise annealing of cortical Arp2/3-nucleated actin bundles (62), supporting the findings of this study. It also should be pointed out that inhibition of actin nucleation during the polymerization process by hindering Arp2/3 complex functions with CK-666 does not result in any apparent changes in the actin cytoskeletal structure or occludin immunostaining pattern in brain endothelial cells and brain microvessels.

Another novel observation in this study involves evidence that METH induces coronin-1b phosphorylation via activation of the MAPK signaling. Coronin-1b, the most widely expressed member of the coronin family, is known to interact and block activity of the Arp2/3 complex (20, 35). Importantly, this negative interaction is inhibited by phosphorylation at serine 2 (Ser-2) on coronin-1b (20, 63). Although protein kinase C (PKC) was suggested to be involved in this process (63), our data indicate that Ser-2 phosphorylation of coronin-1b is mediated by the MAPK pathway in response to METH treatment. However, we also observed that coronin-1b is involved in WASp binding to the Arp2/3 complex after METH exposure. When coronin-1b expression was reduced by the specific siRNA, METH-induced interaction between WASp and Arp2 was diminished. As coronin-1b can also recruit the Arp2/3 complex to the sides of actin filaments (20) and the actin filaments increase the affinity of Arp2/3 complex for the WASp C terminus (64), METH-induced Arp2/3 complex-related actin polymerization might be the outcome of the coordinated works from coronin-1b and WASp.

Along with the previously published study (23), our results suggest that METH-induced small GTPase activity can be a trigger that stimulates WASp activation, followed by activation of the Arp2/3 complex and followed by phosphorylation of ERK1/2. Activated ERK1/2 then mediates phosphorylation of coronin-1b, resulting in further stimulation of Arp2/3 complex activity and Arp2/3 complex-related actin polymerization. These events are important because they directly affect both cellular occludin levels and functional disruption of BBB integrity. Indeed, blocking the Arp2/3 complex formation protected against a METH-induced decrease in occludin levels, transendothelial migration of monocytes, and the increased BBB permeability, suggesting a close interaction among these processes. Our present results indicate that stimulation of actin reorganization can also induce dysfunction of the endothelial barrier integrity via redistribution of cellular occludin.

**FIGURE 6.**
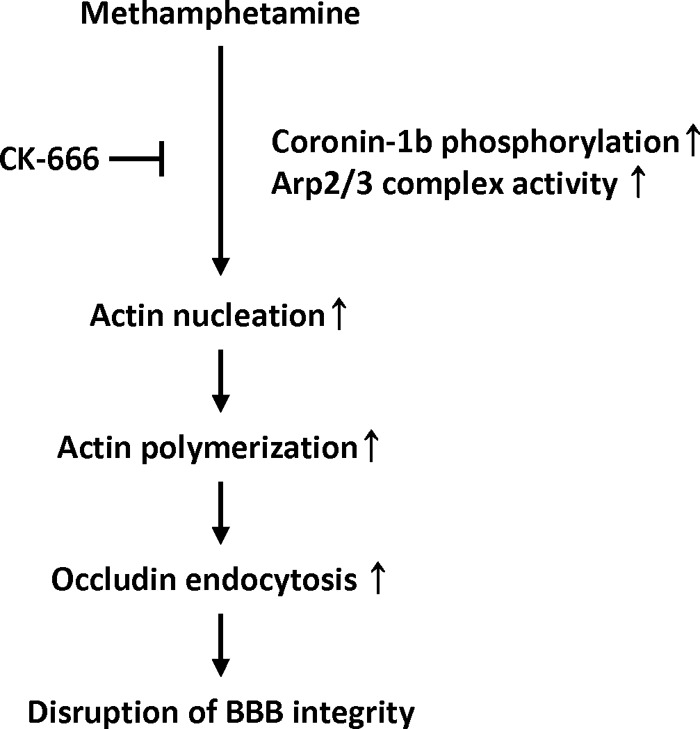
**Proposed involvement of actin rearrangement in METH-induced occludin endocytosis and endothelial barrier dysfunction.** METH induces Arp2/3 complex activation with the subsequent phosphorylation of ERK1/2 and coronin-1b. Phosphorylation of coronin-1b contributes to activation of the Arp2/3 complex. These events stimulate actin nucleation, initiating actin polymerization and resulting in occludin endocytosis. Disruption of the barrier integrity and an increase in transendothelial migration of monocytes appear to be an ultimate outcome of these processes.

In summary, this study demonstrates that exposure to METH induces actin polymerization via activation of the Arp2/3 complex and MAPK-stimulated phosphorylation of coronin-1b. Importantly, these alterations induce occludin rearrangements, functional disruption of the endothelial barrier, and increased transendothelial migration of inflammatory cells (Fig. 6). This study indicates the importance of the actin cytoskeleton in regulating occludin levels and maintaining endothelial integrity.

## Footnotes

3 The abbreviations used are:

METH: methamphetamine
BBB: blood-brain barrier
Arp2/3: actin-related protein 2/3
TJ: tight junctions
WASp: Wiskott-Aldrich syndrome protein
N-WASp: neural-WASp
EEA1: endosome antigen 1
MLC: myosin light chain.
